# Syntheses, Spectroscopic and AFM Characterization of Some Manganese Porphyrins and Their Hybrid Silica Nanomaterials

**DOI:** 10.3390/molecules14041370

**Published:** 2009-03-27

**Authors:** Eugenia Fagadar-Cosma, Marius Constantin Mirica, Ionel Balcu, Carmen Bucovicean, Carmen Cretu, Ileana Armeanu, Gheorghe Fagadar-Cosma

**Affiliations:** 1 Institute of Chemistry Timisoara of Romanian Academy, 24 M. Viteazul Ave, 300223-Timisoara, Romania; 2 National Institute of Research for Electrochemistry and Condensed Matter, Timisoara, 144 Aurel Paunescu Podeanu Street, 300860-Timisoara, Romania; 3 "Politehnica" University of Timisoara, 2 T. Lalescu Street, 300223-Timisoara, Romania

**Keywords:** Porphyrins, Mn(III)-metalloporphyrins, Hybrid silica nanomaterials, AFM, Spectroscopy.

## Abstract

The present work is concerned with the manganese complexes of 5,10,15,20-tetraphenylporphyrin and of 5,10,15,20-tetra(3-hydroxyphenyl)porphyrin, which were prepared by metallation of the corresponding porphyrin ligands, and the study of their spectroscopic and photophysical behavior under strongly acidic and alkaline conditions. The second objective was to obtain and study some new hybrid materials, with special optoelectronic and surface properties, by impregnation of silica gels obtained by one step acid and by two steps acid-base catalysis with these Mn-porphyrins. The resulting nanomaterials exhibited interesting bathochromic and hyperchromic effects of their second band in the emission spectra in comparison with the Mn-porphyrins and also they have distinct orientation of the aggregates on surfaces, as shown by AFM images, making them useful for applications in medicine, formulation of sensors and for environmental-friendly catalysts for photodegradation of organic compounds.

## 1. Introduction

Free-base porphyrins and their metallic complexes hold a special place in modern chemistry functioning as reaction catalysts, oxygen transporters, chemical sensors [1,2], anticancer pharmaceutical drugs [3] or in molecular electronic devices [4]. They are widely distributed in Nature, being also named *pigments of life*, and playing crucial roles in many biochemical processes. In medicine they are suitable for treatments in photodynamic therapy [5], malaria [6] and porphyrias.

The manganese porphyrins represent new molecular designs that achieve redox control [7], while maintaining low molecular weight and tailored lipophilicity. The stability of metalloporphyrins compared to other metal chelates has made them an attractive starting point to develop a new series of Mn(III) porphyrins as superoxide dismutases (SOD) mimetics [8]

Present state-of-the-art research on metal-containing systems of medical importance has developed the design of manganese porphyrins as therapeutic agents and diagnostics in photodynamic therapy (PDT) as well as applications such as potential new tools for electron paramagnetic resonance (EPR) [9], in apoptosis [10], and efficient photosensitizers (with the Q band- λ_max_ = 893 nm- shifted to the near infrared region) [11].

Mn(III)-porphyrins have been used as catalysts in oxidative degradation of Plasmid Bluescript [12] and as ionophores in new potentiometric, piezoelectric or fluorimetric sensor devices for the detection of hydrazine [13] thiocyanate [14] dioxins [15], and salicylate [16].

**Scheme 1:**
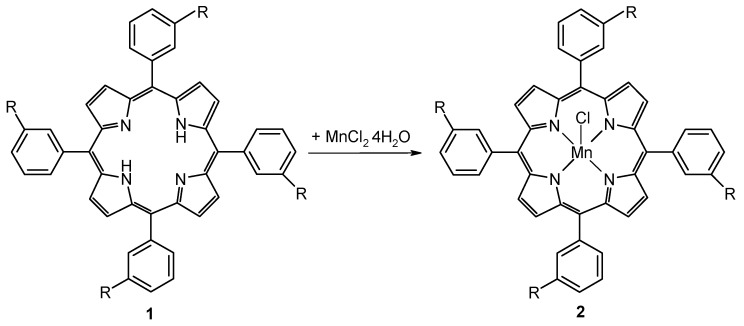
Syntheses of Mn-porphyrins by metallation reactions (**a**, R = H; **b**, R = OH).

The present report is related to previously published work regarding multicomponent syntheses of porphyrins containing *meso*-3-hydroxyphenyl groups [17,18] as potential photosensitizers for photodynamic therapy and deals with the manganese complexes of 5,10,15,20-tetraphenylporphyrin (**1a**) and of 5,10,15,20-tetra(3-hydroxyphenyl)porphyrin (**1b**) with the purpose of using them in cell apoptosis studies and in the design of hybrid nanomaterials with potential applications as new catalysts and potentiometric or enantioselective sensors. The two Mn(III)-porphyrins were prepared by metallation of the porphyrin free ligands in either methanol or ethanol at porphyrin: manganese ratios of 1:20, according to Scheme 1. 

The photophysical behavior of the manganese porphyrins was studied by UV-Vis and emission spectra under strongly acidic and alkaline conditions. The perturbation of pH causes a change in the shape and intensity of the electronic spectra and in the energetic position of the bands. A comparison of the effects produced by the nature of the substituents in the porphyrin ligand was carred out. 

The increasing demand for catalysts in oxidation reactions under mild conditions has provided the necessary impulse for research into new efficient systems based on metalloporphyrin catalysts that mimick natural enzymatic systems. The photoactivity of TiO_2_ anatase samples impregnated with the some Mn-porphyrins was studied [19], and based also on our previous results in obtaining hybrid silica-porphyrin micro- and nano-materials with special optoelectronic properties [20,21,22,23], we have started studying the preparation of some nanomaterials by impregnation of silica gels obtained in one step acid and in two steps acid-base catalysis with these Mn-porphyrins, in order to obtain environmental-friendly catalysts or electroactive materials for sensors design.

## 2. Results and Discussion

*Solubility.* The two manganese(III) porphyrins examined herein are soluble and stable in acetonitrile, *N*,*N*-dimethylformamide, dimethylsulfoxide, dichloromethane, dichloroethane and THF, giving intense green solutions.

*The absorption spectra* of metalloporphyrins are diverse and complex. Depending on the type of donor–acceptor bonds there are three classes of metalloporphyrin electronic spectra, which have been called normal, hypso, and hyper, with the hyper spectra being further divided into p-type and d-type [29,33]. The third type of spectra (d-hyper type) is observed for the Mn(III) complexes of all of the porphyrins studied. Electronic spectra of the hyper type are observed for metalloporphyrins having unoccupied orbitals in metals with symmetries *eg*(*d*(p)-*dxz* and *dyz*. The intense band in the range 440–480 nm in the spectra of the above complexes is assigned to the charge transfer from the *a*1*u*(π) and *a*2*u*(π) orbitals of porphyrin to the *eg*(*d*(π)) orbitals of the metal. The respective band in the spectrum is called the charge transfer band [35]. A comparison between the porphyrin free–bases **1a, 1b** and the two Mn complexes **2a** and **2b** follows. 

**Figure 1:**
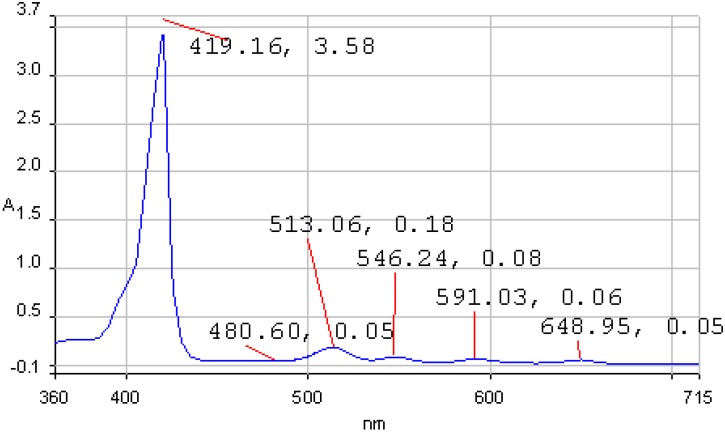
The absorption spectrum of **1a** in THF, c = 3.24×10^-5^M.

**Figure 2:**
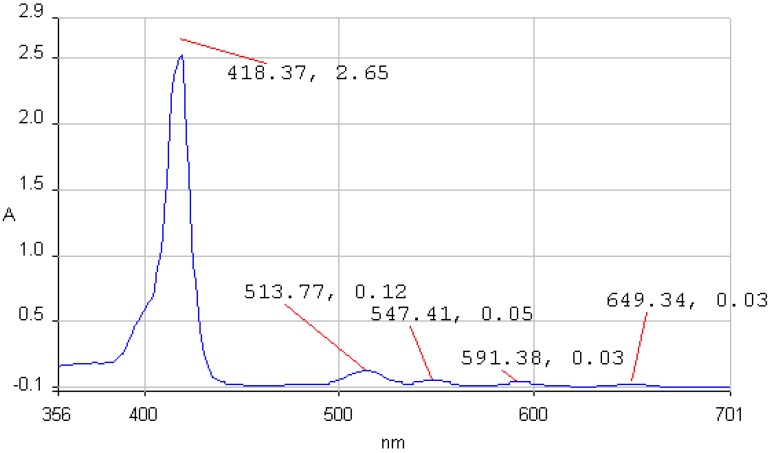
The UV-vis spectrum of **1b** in THF.

The UV-vis spectra of 5,10,15,20-tetraphenylporphyrin (**1a**) and 5,10,15,20-tetrakis(3-hydroxy-phenyl)porphyrin (**1b**) in THF are non-associated (Figure 1 and Figure 2). The spectrum contains the high intensity Soret band, with maximum around 420 nm, followed by four Q bands, Y-polarized bands IV [Q_y(0, 1)_] and III [Q_y(0, 0_)] around 513 and 545 nm respectively, and X-polarized bands II [Q_x(0, 1)_] and I [Q_x(0, 0)_] around 590 and 650 nm, respectively.

**Figure 3:**
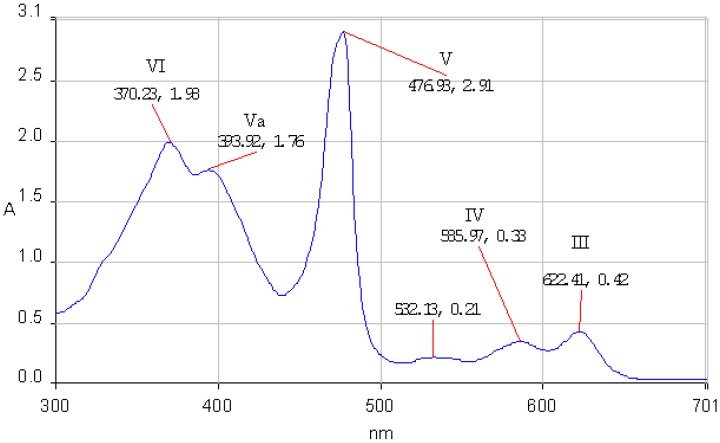
The UV-vis spectrum of **2a** in THF.

**Figure 4:**
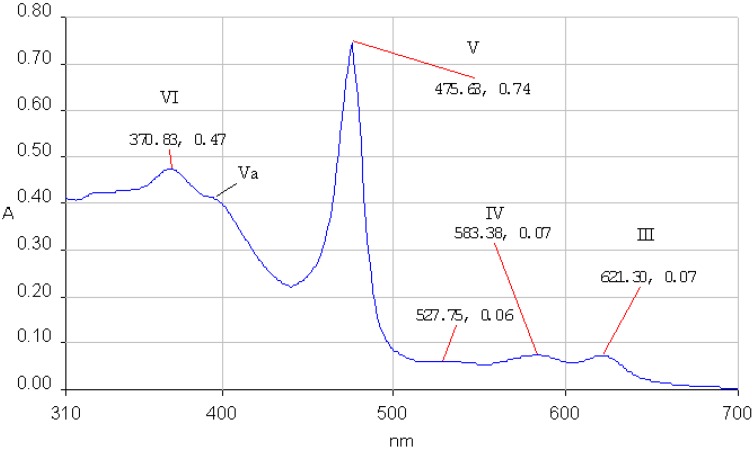
The UV-vis spectrum of **2b** in THF.

According to Boucher [27] the calculated energy levels for the molecular orbitals of a Mn(III) porphyrin are increasing in the following order: a’_2u_(π) < b_2u_(π) < a_1u_(π) < a_2u_(π) < b_2g_(d_xy_) < e_g_(d_xz_, d_yz_) < a_1g_(d_z_^2^) < e_g_*( π) < b_1g_(d_x_^2^-_y_^2^). The UV-Vis spectra of the Mn(III) porphyrins display only two Q bands (III and IV) and the Soret band is in an odd position. Three bands appear between 350-500 nm (V, Va and VI bands). The QI and QII bands cannot be found in the spectrum because they are shifted in the infrared region. The band V, named also Soret band, is strongly influenced by the nature of the counterion and of the axial ligands and arose from a π → π* transition. The e_g_ orbitals of manganese disturbs the π-electron system of the porphyrin producing unexpected positions of the bands in its UV-Vis spectrum. The characteristic absorption bands III, IV, V, Va and VI of the Mn(III)–porphyrin core for **2a** and **2b** in THF are presented in Figure 3 and Figure 4.

**Figure 5:**
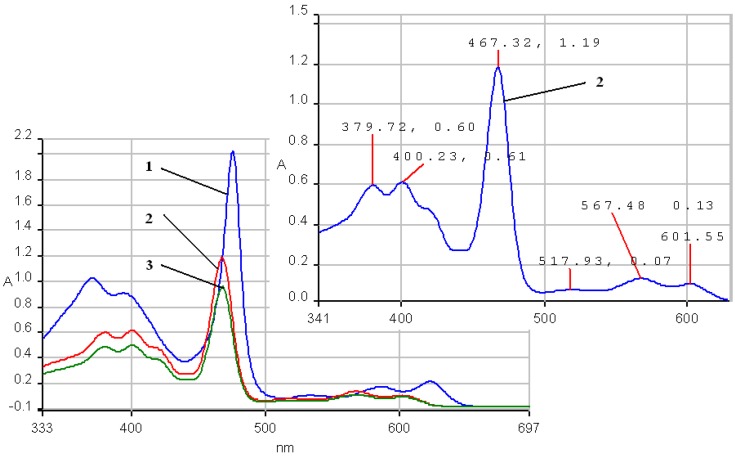
UV-vis spectra. The influence of pH for **2a** in a THF/water system. Curve 1: **2a** in pure THF; curve 2: pH = 1; curve 3: Mn-porphyrin (µ-oxo-dimer) pH = 6.5; Curve 2 is presented in detail.

The stability of the two manganese porphyrins **2a** and **2b** was studied under strongly acidic and alkaline conditions. As the acidity increased (lower pH values; Figure 5), the absorption spectra of the Mn porphyrin complexes in the THF-water system, are characterized by a significant blue shift of the III, IV, V bands and a bathochromic shift of the Va and VI band in comparison with the spectrum in THF. The absorption spectra of the porphyrin complexes **2a** and **2b** in the organic phase were blue shifted with as the pH in the aqueous phase increased, suggesting the formation of the µ-oxo-dimer (Figure 5, curve 3) [28]. Another modest difference to be noticed is that the bands Va and VI show the same absorption intensity.

**Figure 6:**
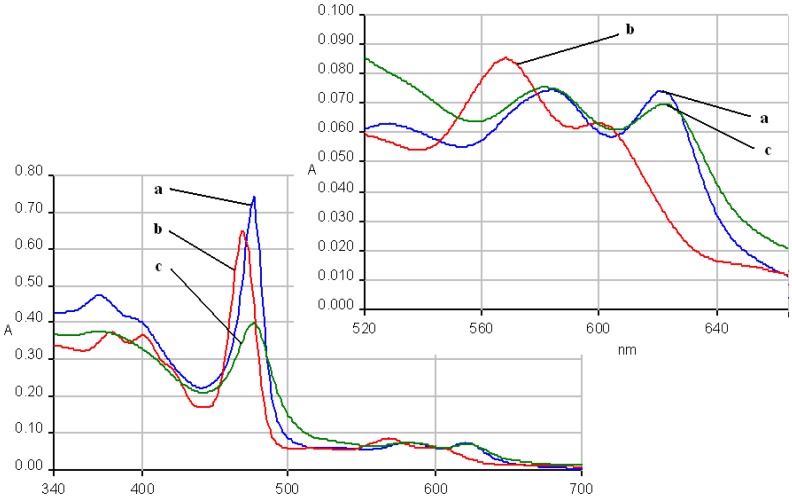
UV-Vis spectra. The influence of pH on the species of **2b** in a THF/water system. Curve a: **2b** in pure THF; curve b: pH = 2.5; curve c: pH = 11. Region of III and IV bands is presented in detail.

The same behavior as in case of **2a** is seen for the electronic spectra of **2b**, accompanying the increasing of acidity band III is hypsochromically shifted by almost 22 nm and band IV is blue shifted by almost 17 nm in comparison with the corresponding band from the spectrum in pure THF and a hyperchromic effect is simultaneously manifested (Figure 6). The band V is also blue shifted by 7 nm but the bands Va and VI show minor bathochromic shifts. By continuously adding base, the shape of the spectrum will coincide with the absorption spectrum in pure THF. The only modest differences consisted of a decrease of the intensity of the V band.

**Figure 7:**
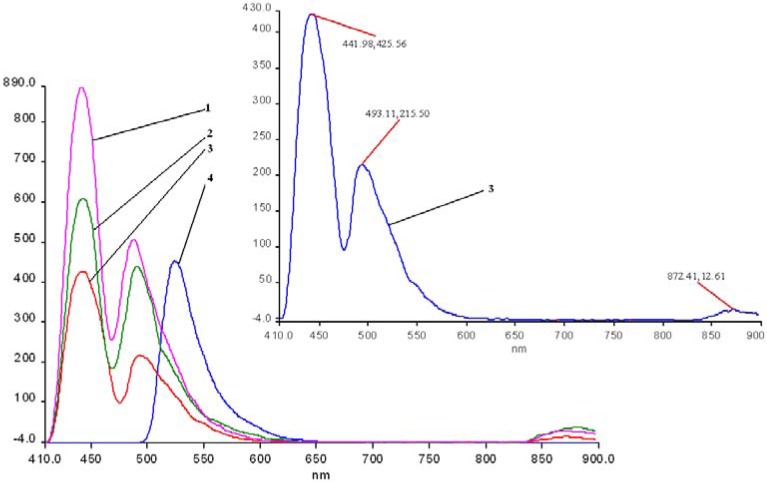
The influence of pH on the emission spectra of **2a** in a THF/water systems (λ_ex_ = 250 nm-filter 430 nm). Curve 1: pH = 1.0; curve 2: pH = 1.5; curve 3: **2a** in THF; curve 4: pH = 11; in detail: curve 3.

**Figure 8:**
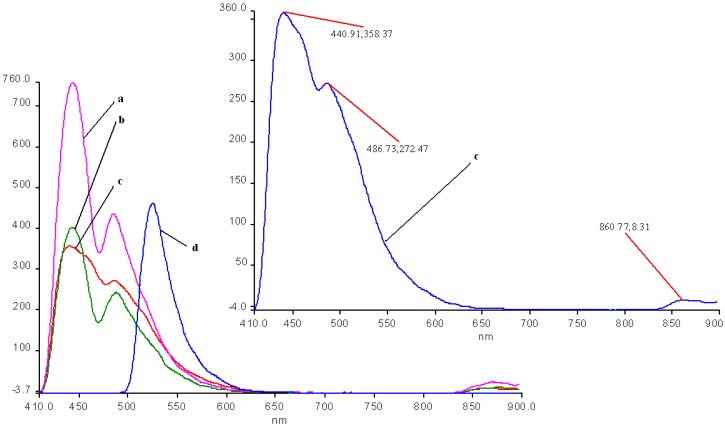
The influence of pH on the emission spectra of **2b** in a THF/water system ( λ_ex_ = 250 nm-filter 430 nm). Curve a: pH = 1; curve b: pH = 1.5; curve c: **2b** in THF; curve d: pH = 11; in detail: curve 3.

The fluorescence emission spectra of **1a** and **1b** registered in THF and THF-water systems at different pH were previously discussed [29,30] and the maximum of the emission bands are around 650-660 nm in pure THF. The fluorescence emission spectra of the manganese (III) porphyrins recorded in THF and THF-water systems, at the same concentration, as the pH conditions vary, are shown in Figure 7 and Figure 8. The spectra of these porphyrins exhibited two distinct maxima, a strong fluorescence band near 440 nm, and an emission band of smaller intensity around 490 nm.

In acidic media, the emission spectra of both manganese porphyrins display a weaker emission band in the 860-873 nm region. All the bands of **2b** are slightly shifted to blue and also hypochromic in comparison with **2a**. 

In basic medium (pH = 12) the shape of the emission spectra of the two Mn complexes **2a**,**b** is completely different, only one almost symmetric band is present, having the maximum of absorption around 523 – 524 nm.

As can be noticed from Figure 7 and Figure 8, a very important increase of the intensity of the emission bands (especially the first band) accompanies the more acidic solutions, but the positions of the bands remain unchanged in comparison with the spectra in pure THF. Instead, a higher value of the pH (basic solution), produces dramatic changes in the shape and position of the emission band and the intensity display only a minor increase, compared with the same spectra in THF.

Another issue is that the bands are well separated in the case of **2a**, but are not well resolved in the caee of **2b**; the cause for this could be a possible aggregation, due to the OH groups.

Based on these spectroscopic data, it can be calculated that negative Stokes shifts, (obtained from the difference between the values of emission band Q(0, 0) and absorption band B(0, 0)) ocurr. Only in the case of a basic pH was a positive Stokes shift of approx. 50 nm observed. The emission from the metal complex occurs with a normal Stokes shift, giving rise to a significant blue shifted emission relative to the protonated species, as explained by some literature data [31]. If large Stokes shifts (180-230 nm) were observed, as other researchers have reported [32], that could suggested that the structure of the porphyrin compound in the excited state is different from that in the ground state, which could indicate a structural reorganization of the porphyrin in the excited state and the distortion of the ligand and porphyrin plane. Moreover, the largest positive Stokes shift can be observed in the case of free porphyrins (230-240 nm), while for manganese complexes the values of Stokes shift are significantly smaller in basic media and negative in the remainder of cases. As other studies have stated [31], a large Stokes shift is favorable for the fluorescence properties because the shift allows easy separation of the emission from scattered light, resulting in minimal of the resonance energy transfer. 

**Figure 9:**
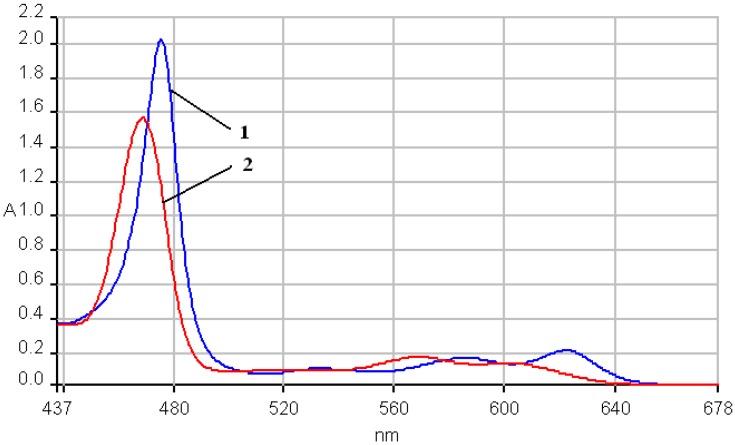
The overlapped UV-vis spectra of **2a** (line 1), and of the hybrid silica material **2a-A** (line 2-pH = 1.5), in THF.

The UV-vis spectrum of the hybrid silica material **2a-A** has all the absorption bands (III, IV and V) hypsochromically shifted in comparison with that of metalloporphyrin **2a** and the V band is wider, suggesting an aggregation process (Figure 9).

**Figure 10:**
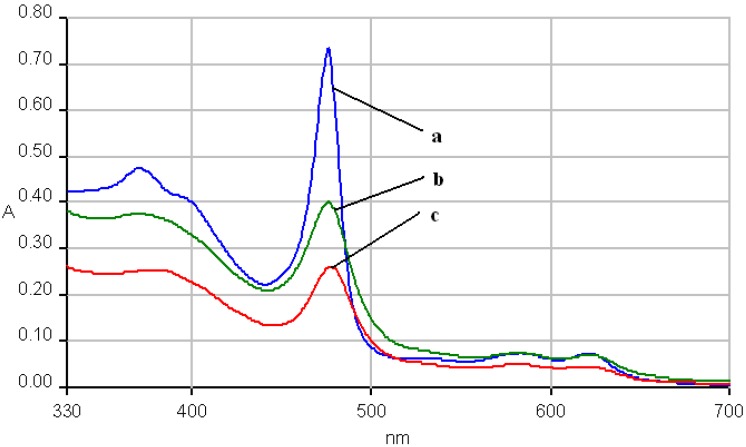
The overlapped UV-vis spectra of **2b** (line a), and of the hybrid silica material **2b-AB** (line b-pH = 11 and line c-pH = 12), in THF.

The electronic spectra of the hybrid silica material **2b-AB** suffered no change regarding the position of the absorption bands, but a significant decrease of the intensity of the absorption (Figure 10).

**Figure 11:**
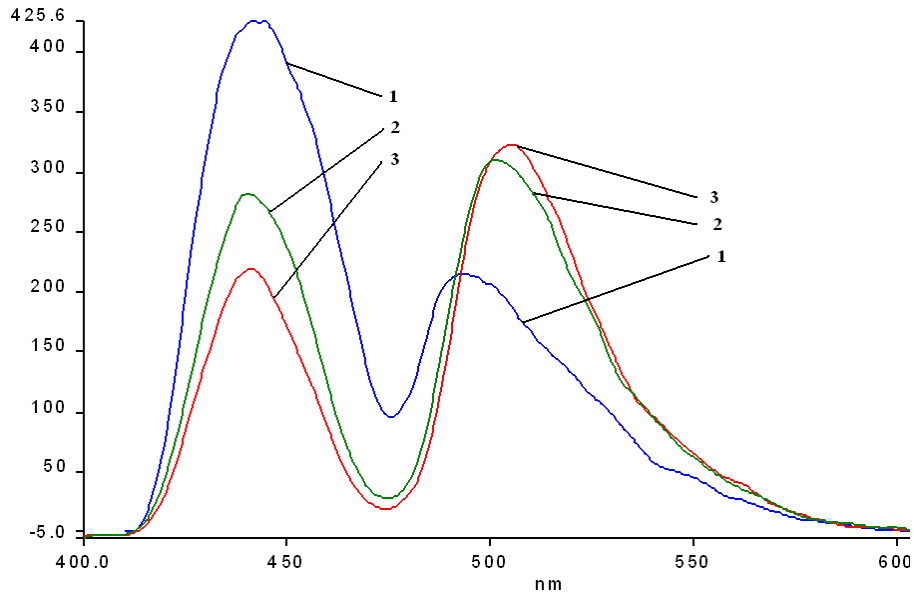
Overlapped emission spectra of hybrid nanomaterials obtained by impregnation of **2a** (line 1) into the silica matrix: **2a-A** (line 2); **2a-AB** (line 3) in THF, at the same concentration.

**Figure 12:**
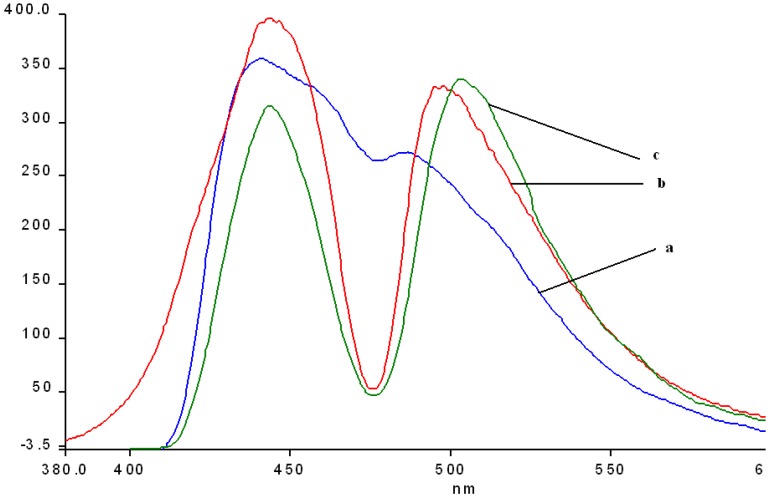
Overlapped emission spectra of hybrid nanomaterials obtained by impregnation of **2b** (line a) into the silica matrix **2b-A** (line b)**; 2b-AB** (line c) in THF, at the same concentration.

The fluorescence spectra of the hybrid silica-Mn-porphyrins **2a-A**, **2a-AB**, **2b-A** and **2b-AB** show some interesting features. In comparison with the emission spectrum of **2a** (Figure 11, line 1), independent of the type of catalysis, both its hybrid-silica materials manifest bathochromic shifts of the two bands of 5 (**2a-AB**) to 15 nm (**2a-A**), but the most interesting aspect worth mentioning is that the first absorption bands around 443-445 nm are hypochromic, but the second bands in the range 501-505 manifest important hyperchromic effects. The same effects are to be mentioned regarding the emission spectra of the hybrid materials obtained by impregnation of **2b** (Figure 12).

**Figure 13:**
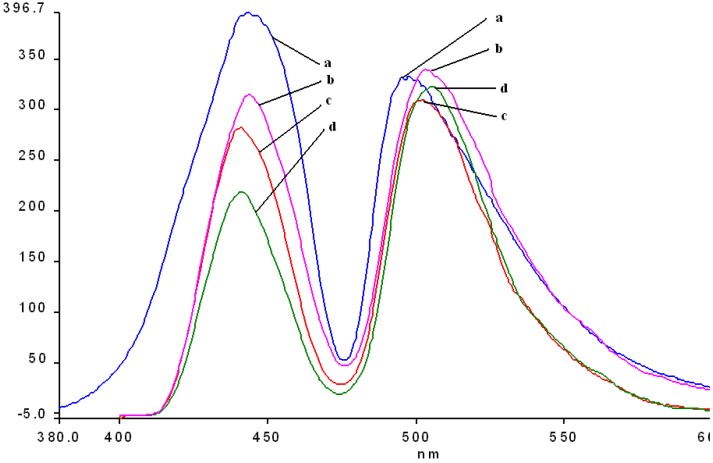
Overlapped emission spectra of hybrid nanomaterials obtained by impregnation of Mn-porphyrins **2a, b** into the silica matrix: line a: **2b-A**; line b: **2b-AB**; line c: **2a-A**; line d: **2a-AB**; in THF, at the same concentration.

**Figure 14:**
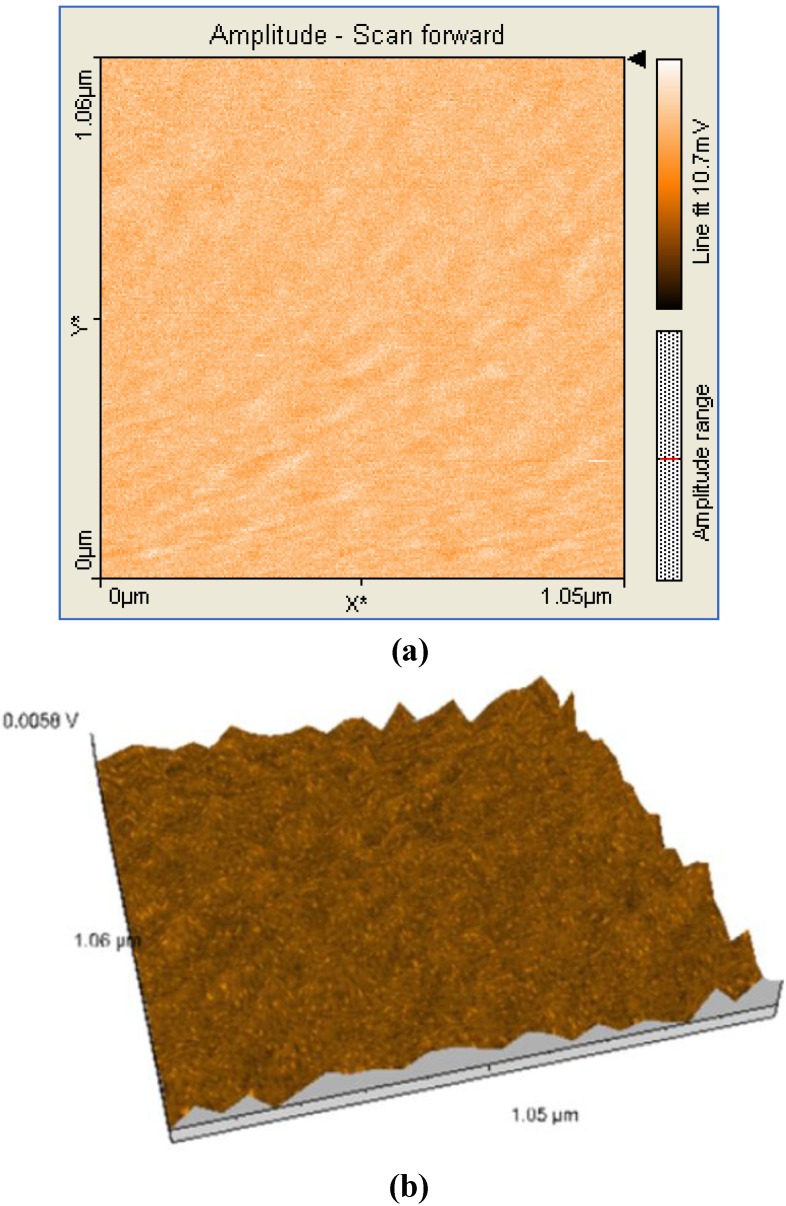
2D (a) and 3D (b) AFM images (1.05×1.06µm) and particle analysis of the hybrid material **1a-AB .**

**Figure 15:**
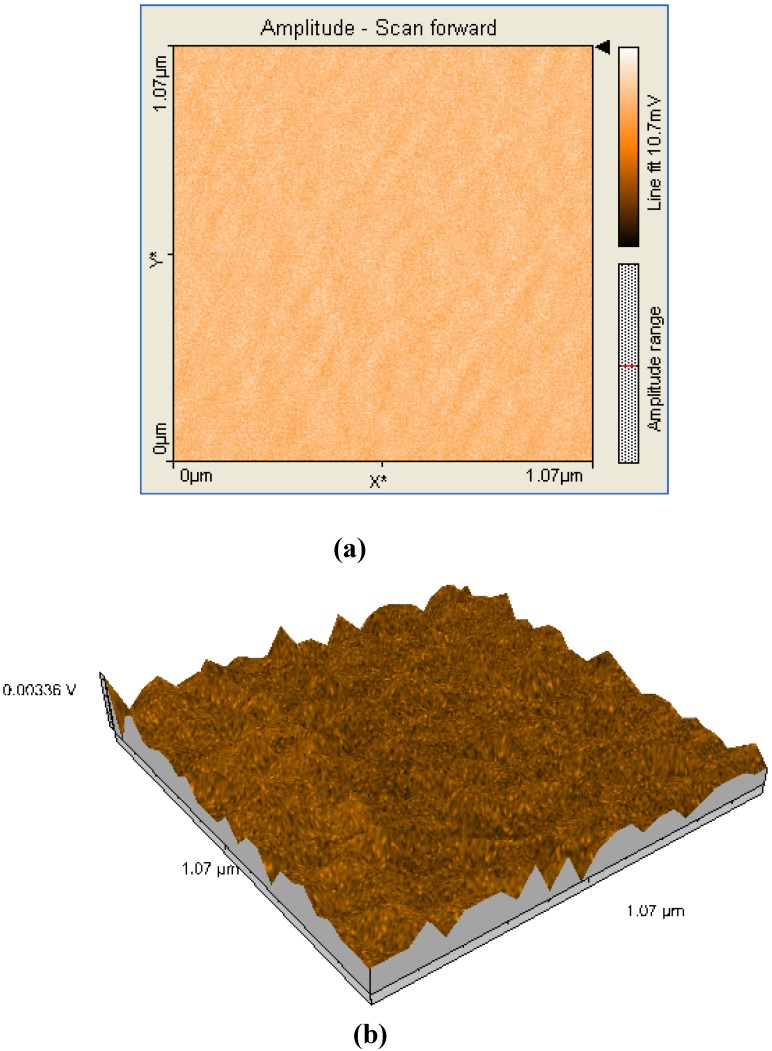
2D (a) and 3D (b) AFM images (1.07×1.07µm) and particle analysis of the hybrid material **1a-A**.

**Figure 16:**
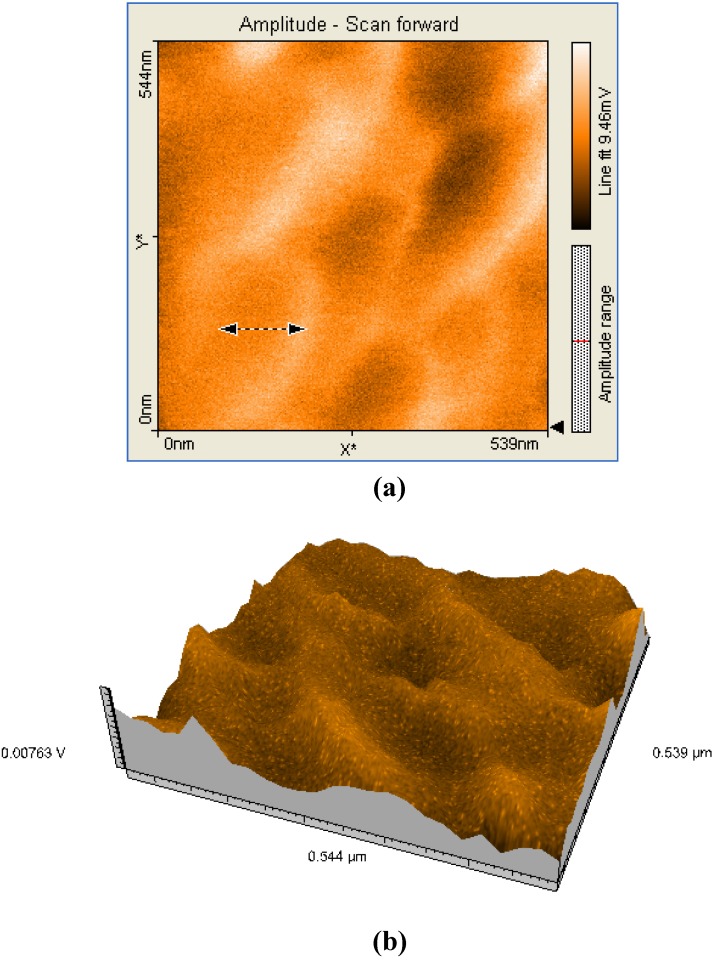
2D (a) and 3D (b) AFM images (539×544nm) and particle analysis of the hybrid material **2b-AB**.

**Figure 17:**
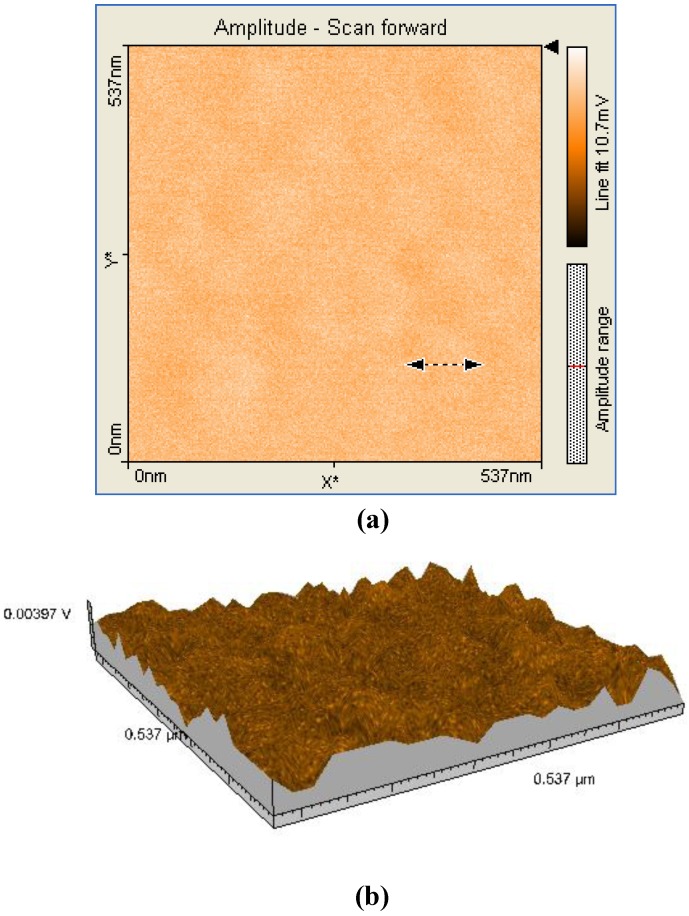
2D (a) and 3D (b) AFM images (537×537nm) and particle analysis of the hybrid material **2b-A.**

A comparison between all the hybrid materials revealed a visible hyperchromic effect when **2b** was used (Figure 13). Atomic force microscopy of the hybrid nanomaterials obtained by impregnation of Mn-porphyrins **2a,b** onto the silica matrix synthesized via a two step acid-base sol-gel procedure or via a one step acid sol-gel procedure (Figure 14, Figure 15, Figure 16 and Figure 17), revealed a dependence of the dimension of particles in direct correlation with the structure of the porphyrin-ligand, not as expected, with the type of catalysis. This odd behavior has not been reported before.

Thus, when silica matrices were impregnated with **2a**, the hybrid nanomaterials **2a-A** and **2a-AB** show surface particles of 54-55 nm in diameter with an average height of 60 nm. In the case of silica matrices impregnated with **2b**, the surfaces of **2b-A** and **2b-AB** present aggregates of 102-122 nm in diameter and 40 nm in height. The aggregates are of increased dimensions compared with the case of **2a** and we assume that the presence of hydroxyl groups and hence of hydrogen bonds resulted in formation of aggregates [18]. Regardless of the nature of the porphyrin ligand, a small increase of the diameter of the particles occurred when silica matrix was obtained by two steps acid-base catalysis. The AFM images shows that these materials have distinct orientation of the aggregates on surfaces, this being an important condition for nanomaterials used in sensing and catalytic processes, to maintain continuity.

## 3. Experimental Section

### 3.1. General

UV-visible spectra of all reported compounds were recorded on a Perkin Elmer Lambda 12 UV/VIS spectrometer in THF and in THF-aqueous systems using a 1 cm quartz cuvette. Fluorescence spectra were recorded in THF and in THF-aqueous systems on a Perkin Elmer Model LS 55 apparatus in a 1 cm cuvette. FT-IR spectra were registered as KBr pellets on a Jasco 430 instrument. The HPLC analysis were performed at ambient temperature on a Jasco apparatus equipped with a Kromasil SI 100 240 x 4 mm 5 μm polar column and a MD 1510 detector. The samples (20 μL) were analyzed at a flow rate of 1 mL/min with hexane as eluent. ^1^H-NMR spectra were registered in CDCl_3_ on a Bruker DRX 400 apparatus at 400 MHz. Proton chemical shifts, expressed in δ (ppm), were internally referenced to the residual proton resonance in CDCl_3_ (δ 7.26). 

For the MS analysis of the porphyrin bases a 212 Varian Finnigan Mat mass spectrometer was used. Mass spectrometric measurements for complexes were performed on a Finnigan LCQ –MS equipped with electrospray ionization source (ESI) and an ion trap mass analyzer in positive ion mode. The samples were dissolved in methanol and filtered through fine membranes (0.4-0.5 micron). Manganese porphyrins gave a molecular ion lacking the chloride counterion.

Tetrahydrofuran (THF) was distilled from sodium/benzophenone, and pyridine was distilled from CaH_2_. Other reagents were provided of highest purity obtainable from Merck and Fluka and were used without further purification. All aqueous solutions were prepared with distilled water. Thin-layer chromatography (TLC) was performed using Merck 60 F254 silica gel. Silica gel 60 (70–230 mesh, Merck) was used for column chromatography.

### 3.2. AFM investigations

AFM investigations were carried out with a Nanosurf^®^ EasyScan 2 Advanced Research AFM. Measurements were made with sample preparation on a silica plate. A stiff (450 μm x 50 μm x 2 μm) piezoelectric ceramic cantilever (spring constant of 0.2 Nm^-1^), with an integral tip oscillated near its resonance frequency of about 13 kHz was used in the measurements. AFM data are quantitative on all three dimensions, the usual method for displaying them being by color mapping: in a gray scale, for example, dark and light tones represent the low, respectively high features. All AFM measurements were done in ambient conditions (temperature: 21±2 °C; relative humidity: 50–70%) in contact mode.

### 3.3. Spectroscopic studies

Absorption and fluorescence spectra were recorded at ambient temperature. All the fluorescence spectra were recorded with constant slit widths, 7 for excitation and 3 for emission. To produce basic and acidic conditions in the THF solvent, stock aqueous solutions of 0.1 M NaOH and 0.1 M HCl were used. The pH values of the solutions were measured with a Radelkis digital pH meter. 

### 3.4. General procedure for the syntheses of porphyrin bases: 5,10,15,20-tetraphenylporphyrin *(**1a**)* and 5,10,15,20-tetra (3-hydroxyphenyl) porphyrin *(**1b**)*.

Porphyrin free bases (**1a**) [33] and (**1b**) [17] were obtained and characterized by methods previously described in the literature [29]. *5,10,15,20-Tetraphenylporphyrin* (**1a**), was fully characterized in a previously published paper [34]. 

*5,10,15,20-Tetrakis(3-hydroxyphenyl)porphyrin* (**1b**): Dark violet solid; yield: 20.4 %; mp > 320°C: ^1^H-NMR: d, ppm: -2.78 (brs, 2H, NH), 2.91 (brs, 4H, OH), 7.20-7.95 (m, 16H, *o*-Ph, *m*-Ph and *p*-Ph), 8.90 (d, 8H, *β*-Pyr); FT- IR (KBr), cm^-1^: 781.99 (γ C-H _Pyrrol_), 1079.94 and 1159.01 (δ C-H _Pyrrol_), 1271.82 (ν C-O-H), 1349.93 (ν C-N), 1445.39 (ν C = N), 1484.92 (ν C = C _Pyrrol_), 1591.95 (ν C = C _Pyrrol_), 3425.92 (ν N-H); UV-vis, CHCl_3_ (λ_max_ (log ε): 421.50 (5.47); 517.87 (4.26); 554.20 (3.91); 591.70 (3.70); 649.05 (3.61); TLC (R_f_ chloroform/dichloromethane/ethylic ether 2/2/1): 0.26; HPLC (R_T_ , min): 7.23; MS (54 eV):m/e = 678 M]^+.^(C_44_O_4_N_4_H_30_]^+.^ Molecular ion).

### 3.5. General procedure for the syntheses of (5,10,15,20-tetraphenyl)porphinato manganese (III) chloride *(**2a**)* and [5,10,15,20-tetra(3-hydroxyphenyl)] porphinato manganese (III) chloride *(**2b**)*.

These syntheses were based on an adaptation of combined literature methods [18,19] and [35]. The free base 5,10,15,20-tetraphenylporphyrin (**1a**, 128 mg, 0.200 mmol) or 5,10,15,20-tetra (3-hydroxyphenyl) porphyrin (**1b**, 140 mg, 0.2062 mmol) in EtOH (40 mL) was heated and stirred and manganese(II) chloride tetrahydrate (816 mg, 4.124 mmol) dissolved in absolute EtOH (10 mL) was then added and the solution was heated to reflux. The reaction was monitored by UV-Vis spectroscopy (solvent THF). The metal insertion was complete in 3 h. The solution was cooled to room temperature under stirring, open to the air and the solvent was evaporated under vacuum, then the crude product was dissolved in dry CH_2_Cl_2_ (400 mL) and a few dark brown powder impurities were removed by filtration. The filtered solution is three times washed with distilled water (70°C) to remove excess MnCl_2_, and the aqueous layer was separated. The organic layer was repeatedly washed with saturated NaHCO_3_ solution, dried over anhydrous Na_2_SO_4_ and filtered. The solvent was removed under vacuum and a dark-green solid of **2a** or **2b** was obtained in 76% or 71% yield, respectively, after crystallization from a 1:1 mixture of THF/CH_2_Cl_2_ or CHCl_3_/hexane or from isopropanol and drying, first under vacuum and then in an oven at 100 °C for 12 h. Better yields were obtained when the solvent was DMF and the MnCl_2_・4H_2_O were dissolved in absolute MeOH. Unlike cobalt(II) tetraphenylporphyrin, solutions of Mn(II)-porphyrins are unstable in the presence of oxygen, and this is the reason why Mn(III) complexes are obtained by the above methods.

*(5,10,15,20-Tetraphenyl)porphinato manganese (III) chloride* (**2a**): dark green crystals, yield: 76 %; mp over 320°C: TLC (SiO_2_, CH_2_Cl_2_/CH_3_OH 8/2) *R_f_* = 0.31. FT- IR (KBr), cm^-1^: 454.15 (ν M-N), 701.96 and 751.13 (γ C-H _Ph_), 803.2 (γ C-H _Pyrrole_), 1007.62 (ν C-N _Pirrole_), 1072.23 and 1174.44 (δ C-H _Ph_), 1340.28 (ν C-N), 1438.6 (ν C=N), 1484.92 (ν C=C _Ph_), 1594.84 (ν C=C _Ph_), 3021.91 and 3051.8 (ν C-H _Ph_). UV-vis, THF, λmax[nm](log ε[M^-1^cm^-1^]): 622.41(4.32), 585.97 (4.21), 476.93 (5.16), 393. 92 (4.94), 370.22 (4.99). MS(ESI^+^): m/z 667.7 [(M-Cl)^+^] (C_44_MnN_4_H_28_]^+.^Molecular ion).

*[5,10,15,20-Tetra (3-hydroxyphenyl)]porphinato manganese (III) chloride* (**2b**): dark green crystals, yield: 71 %; mp over 320°C: TLC (SiO_2_, CH_2_Cl_2_/CH_3_OH 8/2) *R_f_* = 0.4. FT-IR(KBr, cm^-1^): 452.22 (ν M-N), 706.76 (γ C-H _Ph_), 809.95(γ C-H _Pyrrole_), 1005.7 (ν C-N _Pyrrole_), 1078.01 and 1167.69 (δ C-H _Ph_), 1340.28 (ν C-N), 1439.6 (ν C=N), 1587.13 (ν C=C _Ph_), 3061.44 (ν C-H _Ph_). UV-vis, THF, λmax[nm](log ε): 621.30(4.06), 583.38 (4.06), 475.63 (5.09), sh 400, 370.83 (4.89). MS(ESI^+^): m/z 731.6 [(M-Cl)^+^] (C_44_O_4_ MnN_4_H_28_]^+.^ molecular ion).

### 3.6. General procedure for the preparation of the Mn porphyrin-silica hybrid nanomaterials: (5,10,15,20-tetraphenyl)porphinato manganese (III) chloride impregnated in silica matrix derived from one step acid catalysis *(**2a-A**),* (5,10,15,20-tetraphenyl)porphinato manganese (III) chloride impregnated in silica matrix derived from two step acid-base catalysis *(**2a-AB**),* [5,10,15,20-tetra (3-hydroxyphenyl)] porphinato manganese (III) chloride impregnated in silica matrix derived from one step acid catalysis *(**2b-A**)* and [5,10,15,20-tetra (3-hydroxyphenyl)] porphinato manganese (III) chloride impregnated in silica matrix derived from a two step acid-base catalysis *(**2b-AB**)*

The samples were prepared by impregnating silica gel materials obtained by acid or acid –base catalysis with Mn(III)-porphyrins (**2a** or **2b**), according to the following procedure: the corresponding amount of Mn (III)-porphyrin **2a** or **2b** was dissolved in THF (5 mL, to give a 10^-4^ M solution) and the resulting solution was added in one portion under vigorous mechanical stirring to finely ground silica gel (5 g) dissolved in the smallest possible volume of THF, until a viscous colloidal dispersed system was obtained. The mixture was vigorously stirred for 30 min and was left under Parafilm protection until gelation took place. After several days they gave transparent colored gels and finally glasses: red (when using acid-base catalyzed silica matrix, **AB**) and green (when using acid catalyzed silica matrix, **A**).

### 3.7. Common procedures for obtaining the silica matrices derived from one step acid (A*) and two steps acid/base sol-gel processes (AB**), using TEOS as precursor

*A mixture of H_2_O (2.40 g, 0.133 mol) and HCl 37% (0.066 g, 0.67 mmol) was slowly added dropwise under vigorous stirring to a solution of TEOS (6.945 g, 0.033 mol) dissolved into EtOH (6.14 g, 0.133 mol). The following molar ratios were kept constant during the first acidic step: TEOS: EtOH: H_2_O: HCl = 1: 4: 4: 0.02. The silica matrix derived from one step acid has a time of gelation of 7 days.

**The silica matrix derived from a two step acid/base sol-gel process was obtained after the first acid step by using a total amount of 2.04 g solution of NH_3_ 2.5%. A transparent gel was instantly obtained.

## 4. Conclusions

Two manganese complexes of 5,10,15,20-tetraphenylporphyrin and of 5,10,15,20-tetra(3-hydroxy-phenyl)porphyrin were prepared and their spectroscopic and photophysical behavior under strongly acidic and alkaline conditions was investigated. An important goal of this research was the synthesis of new hybrid nanomaterials, exhibiting special optoelectronic and surface properties, by impregnation of silica gels obtained by one step acid and two step acid-base catalysis with these Mn-metalloporphyrins. Depending on the type of catalysis the resulting nanomaterials display different aggregate dimensions and interesting bathochromic and hyperchromic effects of their second bands in the emission spectra in comparison with the Mn-metalloporphyrins. Relying on AFM images, the hybrids have distinct and continuous orientations of the aggregates on their surfaces, thus making them useful for further potential applications in medicine, formulation of sensors and as environmentally-friendly catalysts.
